# Evaluation of Unsaponifiable Fraction of Avocado Oil on Liver and Kidney Mitochondrial Function in Rats Fed a High-Fat and High-Carbohydrate Diet

**DOI:** 10.3390/metabo14080431

**Published:** 2024-08-04

**Authors:** Marcela González-Montoya, Manuel Alejandro Vargas-Vargas, Olin Torres-Isidro, Claudia Isabel García-Berumen, María Guadalupe Cuiniche-Méndez, Alfredo Saavedra-Molina, Julio Cesar Ontiveros-Rodríguez, Hugo A. García-Gutiérrez, Elizabeth Calderón-Cortés, Christian Cortés-Rojo

**Affiliations:** 1 Instituto de Investigaciones Químico-Biológicas, Universidad Michoacana de San Nicolás de Hidalgo, Morelia 58030, MC, Mexico; 1371614e@umich.mx (M.A.V.-V.); 1584209e@umich.mx (O.T.-I.); 0620929h@umich.mx (C.I.G.-B.); 1900220g@umich.mx (M.G.C.-M.); francisco.saavedra@umich.mx (A.S.-M.); hgarcia@umich.mx (H.A.G.-G.); 2 Consejo Nacional de Humanidades, Ciencias y Tecnologías-Instituto de Investigaciones Químico Biológicas, Universidad Michoacana de San Nicolás de Hidalgo, Morelia 58030, MC, Mexico; julio.ontiveros@umich.mx; 3 Facultad de Enfermería, Universidad Michoacana de San Nicolás de Hidalgo, Morelia 58020, MC, Mexico; elizabeth.calderon@umich.mx

**Keywords:** avocado oil, unsaponifiable fraction, high-fat diet, high-carbohydrate diet, metabolic syndrome, mitochondria

## Abstract

High-fat and high-carbohydrate (HF-HC) diets induce metabolic syndrome via mitochondrial dysfunction and oxidative stress. We have previously shown that this may be prevented by avocado oil, a source of bioactive molecules with antioxidant properties. However, it is unknown if these effects are mediated by the unsaponifiable fraction of avocado oil (UFAO). Thus, we tested if this fraction improves glucose metabolism, bioenergetics and oxidative stress in mitochondria from the kidney and liver of rats fed an HF-HC diet. We found that 12 weeks of an HF-HC diet impaired glucose utilization and increased insulin resistance, which was prevented by UFAO administration. The HF-HC diet decreased respiration, membrane potential and electron transport chain (ETC) function in liver and kidney mitochondria. These mitochondrial dysfunctions were prevented by UFAO intake. Unexpectedly, UFAO increased ROS levels in the mitochondria of control animals and did not decrease them in rats with an HF-HC diet; however, UFAO protects liver and kidney mitochondria from iron-induced oxidative stress. These findings suggest that impairments in glucose metabolism and mitochondrial function by an HF-HC diet may be prevented by UFAO, without decreasing ROS generation but protecting mitochondria from oxidative damage.

## 1. Introduction

High-fat and high-carbohydrate (HF-HC) content is a main feature of ultra-processed (UPC) foods, fast food and sugar-sweetened beverages [1]. The convenience and palatability of UPC foods contribute to their widespread consumption, fostering a nutritional environment prone to overconsumption and poor dietary habits [2]. Alarmingly, UPC foods represent more than half of the total dietary energy consumed in high-income countries, such as the USA, Canada and UK, and between one-fifth and one-third of total dietary energy in middle-income countries, such as Brazil, Mexico and Chile [3]. These diets typically lead to an energy imbalance due to the high caloric density of fats combined with the rapid absorption and utilization of simple carbohydrates [4]. The Western diet (WD) is characterized by an HF-HC dietary pattern, and it is strongly linked to chronic low-grade inflammation, which plays a crucial role in the pathogenesis of metabolic syndrome and increased prevalence of chronic degenerative diseases (CDs), including cardiovascular disease and certain types of cancer [5,6] obesity [7], dyslipidemia [8], insulin resistance and type 2 diabetes [9,10], hypertension [11], non-alcoholic fatty liver disease (NAFLD) [12], chronic kidney disease [13], microbiome dysbiosis [14], among others.

The deleterious effects of HF-HC diets on several tissues and organs have been closely linked to mitochondrial dysfunction, consisting of impaired mitochondrial energetic metabolism and reactive oxygen species (ROS) overproduction [15,16,17]. In target organs of diabetes like the liver and kidneys, mitochondrial dysfunction leads to impaired fatty acid oxidation, increased oxidative stress, inflammation, fibrosis and uncontrolled cell death, leading to cellular damage and organ dysfunction [18,19]. 

Nutraceuticals are natural constituents present in food that provide health benefits through the modulation of metabolism and physiological functions. Their consumption is related to a reduction in the risk of suffering CDs [20] by blocking oxidative stress, inhibiting inflammatory mediators and increasing endogenous antioxidant defenses [21].

Avocado oil (AO) has been used as an ingredient in functional foods because of its high concentration of monounsaturated fatty acids and significant amounts of health-beneficial compounds, such as oleic acid, carotenoids, tocopherols and phytosterols. Compared to other oils, avocado oil contains a high proportion of non-saponifiable matter, representing an interesting nutraceutical with high bioactive potential [22]. We have reported that AO improves brain, renal and hepatic mitochondrial function in diabetic, hypertensive and NAFLD rats by preventing the impairment in mitochondrial respiration, membrane potential (*ΔΨ*) and excessive mitochondrial ROS generation [23,24,25,26]. This has been linked to improvements in diabetic and hypertensive nephropathy [27,28], as well as in the development of HF-HC-induced NAFLD [12]. However, it is unknown if these effects were exerted by the unsaponifiable fraction of avocado oil (UFAO). Moreover, food supplements based on UFAO and soybeans or other plant extracts are available commercially [29], but little is known about whether UFAO improves mitochondrial bioenergetic metabolism and oxidative stress in the context of the consumption of an HF-HC diet. Therefore, this study, for the first time, addresses this issue, as UFAO may be a source of molecules with pharmacological actions against the development of metabolic syndrome and related diseases. On this basis, the objective of this study was to determine whether UFAO improves glucose metabolism in rats fed an HF-HC diet and whether this is related to an improvement in energy metabolism and oxidative stress in liver and kidney mitochondria. 

## 2. Materials and Methods

### 2.1. UFAO Preparation

Avocado oil (Chosen Foods, San Diego, CA, USA) was obtained from a local grocery. Unsaponifiable fraction was obtained according to D’Silva and D’Souza [30] with slight modifications. AO (100 mL) in ethanol/water (3:1 *v*/*v*, 400 mL) and KOH (70 g) was refluxed for 2 h to carry out saponification, followed by dilution with equal volumes of water and extraction of UFAO with n-hexane. Hexane was removed using a rotary evaporator (Büchi Labortechnik AG, Flawil, Switzerland) at 45 °C and 345 mbar, and the residue obtained was collected and stored at 4 °C till use. 

### 2.2. AO and UFAO Characterization

Then, 1 µL of AO or UFAO sample was dissolved in 1 mL of chloroform for analyses. Gas chromatograph Bruker Scion SQ 456-GC system (Bruker, Falkenried, Hamburg, Germany) equipped with a CombiPAL autosampler and a Bruker TQ mass detector were used with an RXI-5 SIL Restek fused silica column (Restek, Bellefonte, PA, USA), 0.32 mm ID, 30 m length, and 1.0 µm film thickness. The operation conditions were liquid injection mode, helium carrier gas (1 mL/min), 220 °C injector temperature, 250 °C detector temperature, 250 °C transfer line temperature, ionization EI 70 eV, MS analysis software data deview, library used: NIST. Total run time was 34 min. 

For electrospray ionization (ESI), 1 mg of methanolic extract was resuspended in 1 mL of methanol and diluted 1:100 to avoid clogging of the capillaries and cones. The diluted samples were analyzed using desorption electrospray ionization mass spectrometry (DESI-MS) in negative and positive modes, employing a microOTOF-QII system (Bruker Daltonics, Billerica, MA, USA). A constant flow rate of 8 μL/min was maintained using a Cole Palmer 74900-00-05 syringe pump (Billerica, MA, USA) loaded with 100 μL of the sample. The capillary voltage was set to 2700 V, and nitrogen was used as the drying and nebulizing gas, with a flow rate of 4 L/min (0.4 bar) and a gas temperature of 180 °C. Continuous spectra were collected in an *m*/*z* range of 50–1500, with a total run time of 1 min, a scan time of 10 s and an inter-scan time of 0.1 s, producing six spectra per sample. The mass spectrometer was operated at a resolution of 11,000 (FWHM) at a mass of *m*/*z* 301.9981 in both negative and positive ion modes. The spectrometer was calibrated with an ESI-TOF Tuning mix calibrant (Sigma-Aldrich, Toluca, Edo. de México, México). Finally, precursor ion scans (ms/ms) were performed using negative electrospray ionization (ESI-) with an appropriate set mass. Based on the obtained pattern, the suitable fragments were analyzed using Bruker Compass Data Analysis 4.0 (Bruker Daltonics), which provided a list of possible elemental formulas using the Generate Molecular Formula Editor, as well as a sophisticated comparison of the theoretical isotope pattern with the measured one (σ value) to increase confidence in the suggested molecular formula (Bruker Daltonics Technical Note 008, 2004). The widely accepted accuracy threshold for confirming elemental compositions was set at 5 ppm.

### 2.3. Experimental Design

Male Wistar rats weighing ~370 g were housed in a bioterium with a controlled environment at constant temperature and light/dark cycles of 12 h/12 h. The care and handling of the animals adhered to the guidelines outlined in the Mexican Federal Regulations for the Use and Care of Animals issued by the Ministry of Agriculture (NOM-062-ZOO-1999). This study received approval from the Institutional Bioethics and Biosecurity Committee of the Instituto de Investigaciones Químico-Biológicas, Universidad Michoacana de San Nicolás de Hidalgo (Morelia, Michoacán, Mexico). Sixteen rats were randomly assigned into four groups of four rats each: CTRL group: rats fed with standard rodent chow (5001, Laboratory Rodent Diet, LabDiet, St. Louis, MO, USA); UFAO group: rats fed with standard rodent chow and 100 mg/kg b.w. of UFAO; HF-HC group: rats fed with high-fat–high-carbohydrate diet and 25% fructose in the drinking water; HF-HC + UFAO group: rats fed with high-fat–high-carbohydrate diet, 25% fructose in the drinking water and 100 mg/kg bw of UFAO. The UFAO was dissolved in a 10% gelatin solution previously reported as vehicle [31], while control groups were administrated with the vehicle.

### 2.4. Diet Preparation

HF-HC diet was prepared by mixing equal quantities of rodent chow (5001—Laboratory Rodent Diet, LabDiet, St. Louis, MO, USA) and a dough containing 20% sucrose, 20% lactose, 10% lard, 20% hydrogenated vegetable oil, 2% sodium cholate, 0.4% choline chloride and 0.15% thiouracil [26]. Food was molded and frozen at −20 °C until use. A cold 25% fructose water solution (Kristar—Glucomex, Guadalajara, México) was offered daily ad libitum. The composition of the CTRL and HF-HC diets is shown in Table 1.

### 2.5. Biochemical Parameters

#### 2.5.1. Determination of Biochemical Parameters in Serum

Serum levels of triglycerides, total cholesterol and high-density lipoprotein cholesterol (HDLc) were determined in a Shimadzu UV2550 spectrophotometer (Shimadzu, Kyoto, Japan) using colorimetric kits from Spinreact (Spinreact SA, St. Esteve d’en Bas, Spain) following the manufacturer’s instructions. 

#### 2.5.2. Low-Density Lipoprotein and Very Low-Density Lipoprotein Cholesterol

Low-density lipoprotein (LDLc) and very low-density lipoprotein (VLDLc) levels were estimated using the Friedewald formula [32]. This formula allows for the calculation of LDLc and VLDLc without direct measurement, using the concentrations of total cholesterol, HDL cholesterol and triglycerides obtained from the assays described previously.

### 2.6. Oral Glucose Tolerance Test (OGTT) and HOMA-IR

OGTT was carried out in the 16th week and two days before the sacrifice. After a 12 h overnight fasting and baseline sampling, a 2 g/kg glucose solution was administered orally by gavage, followed by 15-, 30-, 60-, 90- and 120-min collections of blood from the tail. Blood glucose was determined with an Accu-Check^®^ Instant glucometer. Serum insulin concentration was determined with the Rat Ins1 Insulin ELISA Kit (RAB0904, Sigma-Aldrich, St. Louis, MO, USA) according to the manufacturer’s instructions. Homeostasis model assessment of insulin resistance (HOMA-IR) was determined by the following Equation [33]:

HOMA-IR = Fasting blood glucose (mmol/L) × Fasting insulin (mU/L)/22.5(1)

### 2.7. Mitochondrial Isolation

After 12 weeks of treatment, rats were fasted for 12 h and euthanized by decapitation. Mitochondria were isolated by differential centrifugation as previously described by García-Berumen [26] with modifications. The liver and kidney were excised and placed into ice-cold medium 1 containing 220 mM mannitol, 70 mM sucrose, 1 mM EGTA and 2 mM MOPS, pH 7.4. The liver was cut, washed and homogenized in a Potter-Elvehjem homogenizer. The homogenate was centrifuged at 2000 rpm at 4 °C for 10 min. Subsequently, the supernatant was recovered and centrifuged at 7500 rpm at 4 °C for 10 min. The resulting pellet was resuspended with ice-cold medium 2 containing 220 mM mannitol, 70 mM sucrose, 2 mM MOPS and 0.2% BSA, pH 7.4, and centrifuged at 10,000 rpm at 4 °C 10 min. Centrifugations were carried out in a Sorvall RC6+ centrifuge with the SS-34 rotor. Finally, the pellet was re-suspended in 500 μL medium 2. Mitochondrial protein concentration was measured by the Biuret method [34].

### 2.8. Mitochondrial Function

#### 2.8.1. Oxidative Phosphorylation (OXPHOS) Assessment

OXPHOS was evaluated according to the protocol previously reported [26] by measuring mitochondrial respiration in oligomycin-induced state 4 (state 4) and phosphorylating state (state 3). Oxygen consumption rate (OCR) was determined in freshly isolated mitochondria using a Clark-type electrode coupled to a YSI 5300A biological oxygen monitor and connected to a computer for data acquisition. Then, 0.3 mg/mL of mitochondrial protein was placed into a sealed glass chamber containing respiratory buffer (100 mM KCl, 10 mM HEPES, 3 mM KH_2_PO_4_ and 3 mM MgCl_2_, pH 7.4). The final volume was adjusted to 3.3 mL. Respiration traces were started after adding mitochondrial protein and 10 mM glutamate/malate as a complex I-linked respiratory substrate, and OCR was followed for 90 s. Then, state 3 respiration was stimulated with 0.2 mM ADP, and OCR was measured for 90 s. State 4 was induced by adding 1.4μg/mL oligomycin, and OCR was further recorded for 90 s. Respiratory control ratio (RCR) was calculated by dividing the respiration rate in state 3 and the respiration rate in state 4. Non-mitochondrial respiration was discriminated via the addition of 5 µg antimycin A.

#### 2.8.2. Determination of Mitochondrial Membrane Potential

*ΔΨ* was estimated by a spectrofluorometric assay using safranine-O at λ_ex_ 495 nm and λ_em_ 586 nm in a Shimadzu RF301PC spectrofluorometer [23]. Freshly isolated mitochondria from liver (1 mg/mL) or kidney (0.5 mg/mL) were resuspended in a quartz cuvette containing 2 µM safranine-O in respiratory buffer at a final 2 mL volume. Basal fluorescence was recorded for 1 min. Then, mitochondria were energized by adding 10 mM glutamate/malate and the changes in fluorescence were recorded for 3 min. Finally, the uncoupler carbonyl cyanide 3-chlorophenylhydrazone (CCCP) was added to dissipate *ΔΨ*. 

#### 2.8.3. Determination of ETC Complexes Activities

Complex I activity was assayed in mitochondria permeabilized with osmotic shock, as described in [27]. The activity was evaluated spectrophotometrically following the NADH oxidation in the presence of K_3_Fe(CN)_6_ as electron acceptor. For complex II–complex III activity, antimycin A sensitive cytochrome *c* reduction was spectrophotometrically recorded at 550 nm in mitochondria permeabilized with freeze/thaw cycles in the presence of 10 mM succinate as substrate [35].

### 2.9. Mitochondrial Oxidative Stress

#### 2.9.1. ROS Levels Estimation

Mitochondrial ROS levels were estimated by measuring the oxidation of the fluorescent probe 2′,7′-dichlorodihydrofluorescein diacetate (H_2_DCF-DA) according to a previous report [24] with some modifications. Freshly isolated mitochondria from liver (1 mg/mL) or kidney (0.5 mg/mL) were resuspended in 2 mL respiratory buffer and incubated with 1.25 mM H_2_DCF-DA at 4 °C for 20 min with constant shaking. The assay started by recording basal fluorescence for 1 min. Then, 10 mM glutamate/malate was added as a substrate and changes in fluorescence were recorded for 10 min. The rate of H_2_DCF-DA oxidation, which is proportional to the rate of ROS generation, was estimated by subtracting the final fluorescence obtained after 10 min glutamate/malate addition minus the initial fluorescence detected before glutamate/malate addition and divided by the time of recording and protein concentration.

#### 2.9.2. Lipid Peroxidation Assay

This determination was carried out only in liver and kidney mitochondria from the CTRL and UFAO groups to evaluate whether UFAO intake increases the ability of mitochondria to cope with in vitro induced oxidative damage. Mitochondria (0.5 mg/mL) were resuspended in 1 mL of 50 mM KH_2_PO_4_ buffer (pH 7.6) and incubated for 30 min with increasing concentrations of Fe^2+^ to induce oxidative stress. Malondialdehyde (MDA) and 4-hydroxyalkenals formation was evaluated with a method consisting of the reaction under acidic conditions of these final products of lipid peroxidation with 1-methyl-2-phenylindole, yielding a stable chromophore that was spectrophotometrically detected at 586 nm in a Shimadzu UV2550 spectrophotometer [36].

### 2.10. Statistical Analyses

Results are expressed as the mean ± standard error of at least 4 independent experiments, using samples from different animals for each experiment. For the comparison between more than two groups, statistical differences between means (*p* < 0.05) were determined by analysis of variance (ANOVA), followed by Holm Sidak’s post hoc test. The Holm Sidak test was selected over other tests because it allows testing for multiple comparisons using datasets with varying sample sizes and better controls type I errors (i.e., false positives) while maintaining statistical power [37]. For comparisons between only two means, Student’s *t*-test was used. Statistical analyses were performed with Sigma Plot 14.5 (Systat Software Inc., Palo Alto, CA, USA). The plots showing the individual data points were produced with GraphPad Prism 8.0 (GraphPad Software Inc., San Diego, CA, USA).

## 3. Results

### 3.1. Identification of Compounds in AO and UFAO

The fatty acid composition of AO was assessed by GC-MS (Table 2). The presence of oleic acid (59%) was observed as the main fatty acid, followed by linoleic acid (16.5%). UFAO composition was analyzed by ESI and GC-MS/MS. The presence of an important amount of saturated aliphatic compounds was observed (Table 3), such as decane and derivatives, undecane, dodecane and tridecane, all of them constituting more than 70% of the total composition.

Moreover, compounds identified in UFAO by ESI (Table 4) showed higher molecular weight compared to saturated aliphatic compounds. Compound **1** is an aromatic acid retinoid with multiple methyl groups. Compound **2** is an iridoid glycoside. Compound **3** is a non-carotenoid that presents a chemical structure very close to compound **2**, also with the presence of the glucopyranose moiety. Compound **4** is a derivative of xanthone, highly functionalized with methoxy groups. Compound **5** is a specific type of xanthophyll, a carotenoid pigment that provides yellow or red coloration in various plant foods. Finally, compound **6** is a long-chain diester of a pyranose derivative. The chemical structures of all compounds are depicted in Supplementary Figure S1.

### 3.2. Effect of UFAO on Body Weight, Biochemical Parameters and Glucose Metabolism

We evaluated fasting serum lipids levels at the end of treatments. Cholesterol levels in the HF-HC and HF-HC+UFAO groups were higher compared to that of the CTRL group (Table 5), while there were no differences between the UFAO and CTRL groups. No statistical differences were observed between groups regarding HDL levels (Table 5). The levels of triglycerides and VLDL underwent an increase only in the UFAO group. LDL levels decreased significantly in the HF-HC+UFAO group (*p* < 0.05).

**Figure 1:**
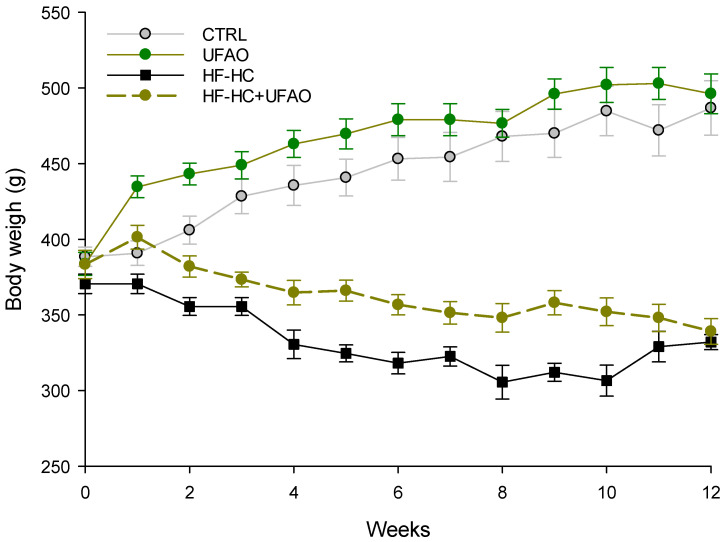
Effect of UFAO on body weight. Values are represented as mean ± standard error of *n* = 4.

The bodyweight at the end of the treatments is depicted in Figure 1. There were no statistically significant differences between the HF-HC and HF-HC+UFAO groups, despite that, between weeks 1 and 10, the HF-HC+UFAO group had higher body weight than the HF-HC group. No significant differences were observed between CTRL and UFAO groups.

**Figure 2:**
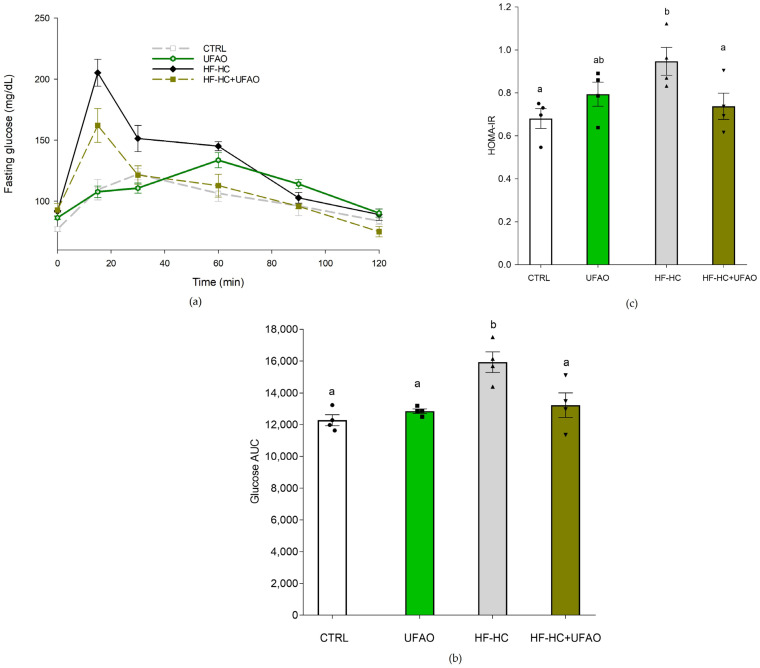
(**a**) Effects of UFAO on oral glucose tolerance test (OGTT), (**b**) area under the curve (AUC) of OGTT (**c**), HOMA-IR. The results are shown as the mean ± standard error of *n* = 4. Different letters indicate statistically significant differences between means at *p* < 0.05 (one-way ANOVA, Holm–Sidak post hoc test).

We determined oral glucose tolerance (OGTT) and insulin resistance. The HF-HC group exhibited the highest levels of circulating glucose during the OGTT at 15, 30 and 60 min. In contrast, the HF-HC+UFAO group had lower glucose levels compared with the HF-HC group, particularly at 15 min. These effects were corroborated when the area under the curve (AUC) (Figure 2b) of glucose was calculated from the data in Figure 2a. The HF-HC group showed the highest AUC, while CTRL, UFAO and HF-HC+UFAO showed similar AUC without statistically significant differences. The HF-HC group had a higher HOMA-IR index in comparison to CTRL (Figure 2c). However, the HF-HC+UFAO group displayed a HOMA-IR index similar to that of the CTRL group. Together, these results indicate that UFAO was effective in improving insulin sensitivity and glucose utilization in rats consuming the HF-HC diet. 

### 3.3. Effects of UFAO on Liver and Kidney Mitochondrial Function

**Figure 3:**
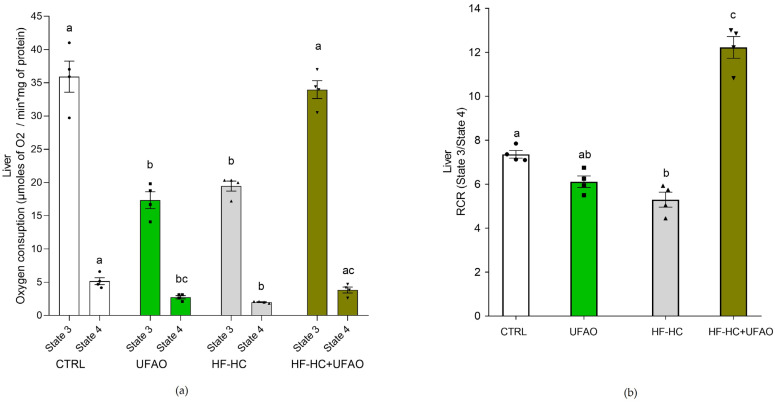
(**a**) Effect of UFAO on respiration rates and (**b**) RCR in liver mitochondria. The results are shown as the mean ± standard error of *n* = 4. Different letters indicate statistically significant differences between groups in the same respiratory state at *p* < 0.05 (one-way ANOVA, Holm–Sidak post hoc test).

**Figure 4:**
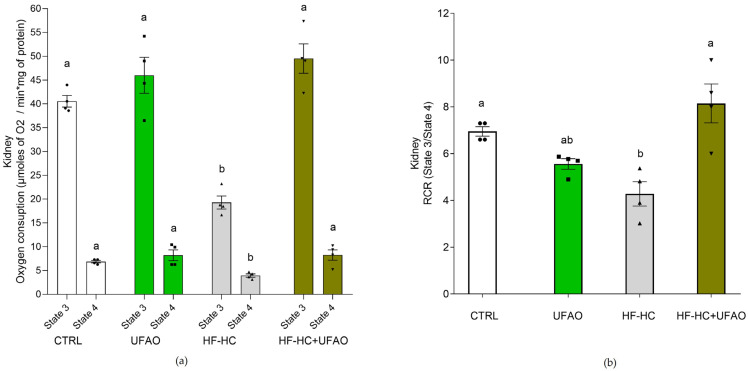
(**a**) Effect of UFAO on respiration rates and (**b**) respiratory control ratios RCR in kidney mitochondria. The results are shown as the mean ± standard error of *n* = 4. Different letters indicate statistically significant differences between groups in the same respiratory state at *p* < 0.05 (one-way ANOVA, Holm–Sidak post hoc test).

OXPHOS was severely affected by the HF-HC diet in liver and kidney mitochondria, as reflected by a decrease in respiration in states 3 and 4 (Figure 3a and Figure 4a, respectively) and RCR (Figure 3b and Figure 4b) in mitochondria in the HF-HC group. Conversely, respiratory states and RCR were similar between the HF-HC+UFAO and CTRL groups in liver and kidney mitochondria (Figure 3 and Figure 4), suggesting an improvement in mitochondrial function in the rats consuming the HF-HC diet supplemented with the UFAO. Of note, compared to mitochondria in the CTRL group, liver mitochondria in the UFAO group exhibited a decrease in respiration rate in both states 4 and 3 (Figure 3a), although non-significant differences in RCR values were observed between these groups (Figure 3b). 

**Figure 5:**
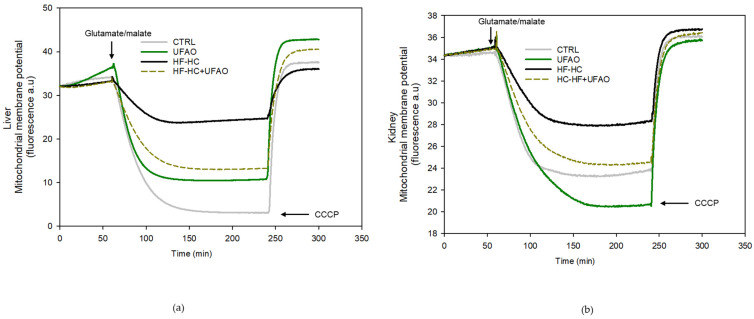
Effect of avocado oil on mitochondrial membrane potential (*ΔΨ*) using glu/mal as substrate. Representative traces of mitochondria from (**a**) liver and (**b**) kidney. Fluorescence was expressed in arbitrary units (a.u.). The traces are representative of *n* = 4.

Regarding *ΔΨ* (Figure 5), the energization of mitochondria in the CTRL group with glutamate/malate elicited an instantaneous and significant decrease in safranin O fluorescence (gray line), which reflects the establishment of the *ΔΨ* in both liver and kidney mitochondria. A decrease in *ΔΨ* was observed in the HF-HC group in liver and kidney mitochondria, as reflected by a lower drop in fluorescence in response to the respirator substrate (black line). *ΔΨ* was improved in the HF-HC+UFAO group (dotted green line), reflected as a more significant drop in safranine-O fluorescence than that in the HF-HC group. The UFAO group did not exhibit apparent alterations in *ΔΨ* establishment, as displayed in the green traces. Together, these results suggest that UFAO prevents an impairment in mitochondrial function in rats consuming an HF-HC diet and that UFAO administration to healthy, control rats only decreases the rate of mitochondrial respiration without impairing the ability of mitochondria to respond to ATP demand (i.e., the RCR) and the establishment of *ΔΨ*.

**Figure 6:**
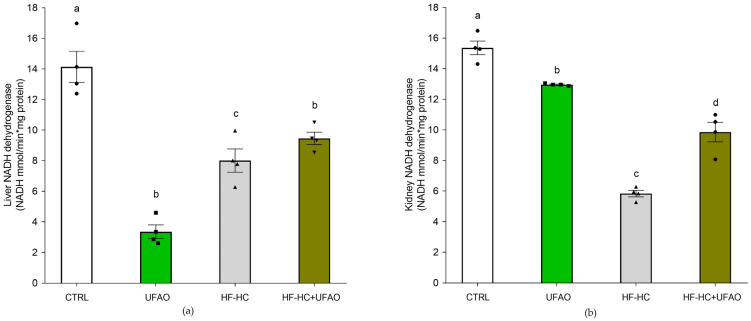
Effects UFAO on the activity of complex I of liver (**a**) and kidney (**b**) mitochondria from rats fed with HF-HC diet. The results are shown as the mean ± standard error of *n* = 4. Different letters indicate significant differences between groups at *p* < 0.05 (one-way ANOVA, Holm–Sidak post hoc test).

**Figure 7:**
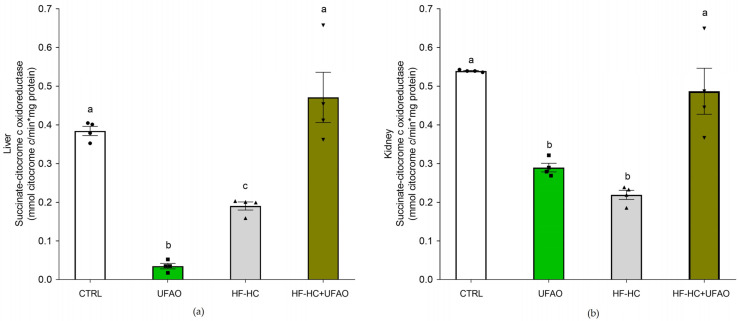
Effects UFAO on the complex II–complex III activity of liver (**a**) and kidney (**b**) mitochondria from rats fed with HF-HC diet. The results are shown as the mean ± standard error of *n* = 4. Different letters indicate significant differences between groups at *p* < 0.05 (one-way ANOVA, Holm–Sidak post hoc test).

The effects of UFAO on ETC function are shown in Figure 6. Complex I activity decreased by more than 70% in liver (Figure 6a) and kidney (Figure 6b) mitochondria in the HF-HC group compared to the CTRL group. A partial recovery in complex I activity was observed in the mitochondria of both organs in the HF-HC+UFAO group. The UFAO group also showed a decrease in complex I activity but of a lower magnitude than in the HF-HC group, reaching the levels of the HF-HC+UFAO group. A similar behavior was observed in complex II–complex III activity in liver (Figure 7a) and kidney (Figure 7b) mitochondria, although UFAO had a more substantial inhibitory effect than in complex I, mainly in liver mitochondria (Figure 7a).

### 3.4. Effect of UFAO on Mitochondrial Oxidative Stress 

**Figure 8:**
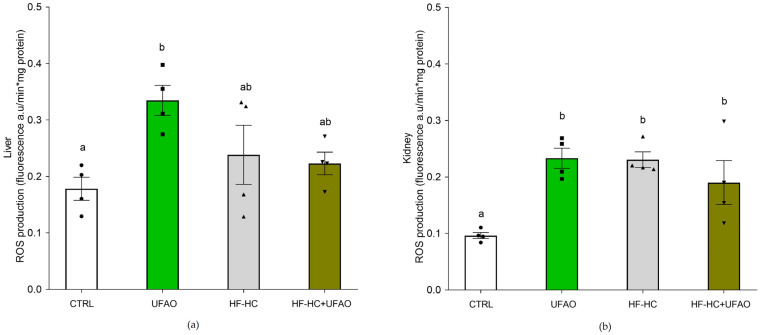
Effect of UFAO on ROS levels in (**a**) liver and (**b**) kidney mitochondria. Results are shown as the means ± standard error of *n* = 4. Different letters indicate statistically significant differences between means at *p* < 0.05 (one-way ANOVA, Holm–Sidak post hoc test).

**Figure 9:**
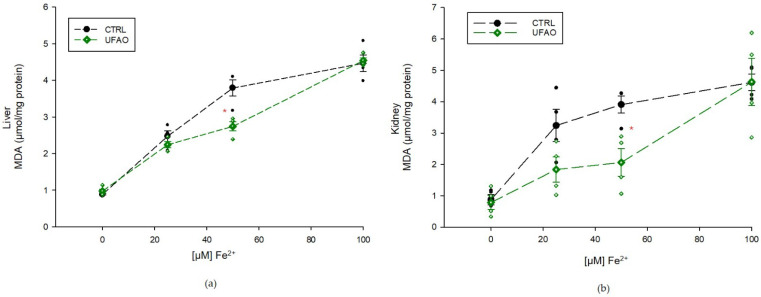
Antioxidant effect of UFAO on (**a**) liver and (**b**) kidney mitochondria with different concentrations of Fe^2+^ measured by MDA production. Results are shown as the means ± standard error (*n* = 4). The asterisk indicates a statistically significant difference between groups at *p* < 0.05 (Student’s *t*-test).

Figure 8 shows the ROS production in liver and kidney mitochondria. In liver mitochondria (a), there were no statistical differences between CTRL, HF-HC and HF-HC+UFAO groups; in contrast, ROS levels in the UFAO group increased in comparison with all the other groups. In kidney mitochondria (b), a higher ROS production was observed in the HF-HC, HF-HC+UFAO and UFAO groups compared to CTRL. Regarding lipid peroxidation (Figure 9), a protective effect was observed in the UFAO group in liver and kidney mitochondria, particularly at 50 µM Fe^2+^.

## 4. Discussion

AO is rich in lipid-soluble bioactive compounds, including unsaturated fatty acids and bioactive lipids [38,39]. These phytochemicals have beneficial effects in various diseases [40]. We identified saturated aliphatic compounds in UFAO, primarily undecane, dodecane and tridecane. Previous metabolomic profiling of four avocado varieties also reported the presence of these hydrocarbons using GC-MS [41]. Undecane possesses anti-inflammatory, antiallergic and immunosuppressant effects, as well as antioxidant and metal chelation activities [40,42]. We did not identify other bioactive compounds previously reported in AO, such as chlorophylls, carotenoids and sterols, which possess antioxidant, anti-inflammatory and hypolipidemic effects [43]. These bioactive compounds are labile at 100–180 °C [22]. The saponification conditions used in this work included boiling temperature for 2 h, and, also, we used a non-polar solvent for the extraction process, which may explain the absence of these molecules in UFAO. 

High-molecular-weight compounds were found by ESI. Compound **3** (Table 4) could be related to benzofuranones, and it was previously described that the 3*H*-benzofuran-2-ones are a significant class of heterocyclic molecules, highly widespread in nature, consisting of a benzene ring fused with a furan-2-one ring, which has exhibited antioxidant, radical scavenging and inflammatory properties, as well as inducible NO synthase (iNOS) expression and platelet aggregation inhibition [44]. Tangeraxanthin (compound **5**) is an aromatic triterpenoid. Xanthines like astaxanthin have been reported to improve hepatic damage and mitochondrial dysfunction in NAFLD [45]. Therefore, there is a possibility that tangeraxanthin mediates the beneficial effects of UFAO against the decrease in OXPHOS (Figure 3 and Figure 4), *ΔΨ* (Figure 5) and CTE complex activities (Figure 6 and Figure 7) induced by the HF-HC diet.

On the other hand, the monitoring of body weight revealed intriguing differences among groups. Notably, the HF-HC group exhibited a lower body weight compared to the HF-HC+UFAO groups during weeks 1 to 10. This unexpected weight loss in the HF-HC group could be indicative of metabolic disturbances often seen in conditions such as diabetes, where HF-HC diets can lead to weight loss due to impaired increased energy expenditure and nutrient utilization [46,47]. The latter is in agreement with the decrease in glucose utilization observed in Figure 2a. In contrast, the HF-HC+UFAO group maintained higher body weight, suggesting that the UFAO may play a role in improving metabolism under these metabolic stress conditions, as reflected by the normalization of the area under the curve of the glucose tolerance curve (Figure 2b) and the improvement in the HOMA-IR index (Figure 2c). No statistical differences in body weight were observed between the CTRL and HF-HC+UFAO groups, indicating that UFAO does not influence body weight in the absence of the HF-HC diet. These findings highlight the potential of UFAO to mitigate some adverse effects of HF-HC diets, possibly through mechanisms that enhance nutrient utilization and reduce metabolic inefficiencies.

HF-HC diets disrupt glucose metabolism, leading to impaired glucose tolerance and insulin resistance [48]. This is consistent with the glucose intolerance (Figure 2a) and increased insulin resistance (Figure 2b) observed in the HF-HC group. In turn, impaired glucose metabolism has been linked to the decreased mitochondrial oxidation of metabolic substrates, culminating in the intracellular accumulation of lipids that interfere with insulin signaling [49]. In addition, the liver is an important contributor to this mechanism of insulin resistance [50,51]. All this is consistent with the decrease in liver mitochondria of respiration rates in states 4 and 3 (Figure 3a), *ΔΨ* (Figure 5a), ETC complexes (Figure 6a and Figure 7a) and the increase in insulin resistance in the HF-HC group (Figure 2c). Importantly, a decrease in mitochondrial respiration has been detected prior to the onset of type 2 diabetes, which is consistent with our data, given that, as seen in Figure 3 and Figure 4, rats in the HF-HC group did not exhibit fasting hyperglycemia but presented impairments in glucose metabolism (Figure 2). On the other hand, it has been observed that a high-fat diet causes renal damage, oxidative stress and mitochondrial dysfunction in kidneys [52]. Thus, the HF-HC diet used in this study could induce renal damage, since impairments in the respiratory chain and oxidative phosphorylation (Figure 4b) were also observed in kidney mitochondria in the HF-HC group. 

Improved glucose metabolism was observed in rats in the HF-HC+UFAO group, as they presented control-like levels of glucose tolerance (Figure 2a) and HOMA-IR (Figure 2c), indicating that UFAO prevented insulin resistance generated by an HF-HC diet. This could have been the result of increased substrate oxidation in mitochondria, leading to less accumulation of incomplete products of fatty acid oxidation such as diacylglycerol and ceramides and, therefore, less interference of the HF-HC diet with insulin signaling. In support of this idea, it was observed that in liver mitochondria, the HF-HC+UFAO group exhibited similar rates of mitochondrial respiration (Figure 3a) and oxidative phosphorylation (Figure 3b) to those of mitochondria from the CTRL group. This, in turn, could be the result of enhanced electron transfer in the ETC, as shown by the enhanced activities of complexes I and II-III in rat liver mitochondria from the HF-HC+UFAO group (Figure 6a and Figure 7a).

In previous studies, we found that avocado oil supplementation prevents the development of nephropathy in diabetic rats and NAFLD in HF-HC diet rats, which was associated with an improvement in the activity of ETC complexes and OXPHOS [26,53]. Therefore, it is feasible to propose that UFAO components may have been responsible for the beneficial effects of avocado oil on NAFLD- and diabetes-induced liver and kidney damage, respectively. Thus, there is a possibility that UFAO supplementation may delay the development of hepatic and renal complications of metabolic syndrome once insulin resistance is established prior to the development of diabetes. However, further studies in models of diabetes and NAFLD with UFAO remain to be conducted to corroborate this hypothesis. 

Conflicting results were obtained with respect to ROS production and lipid peroxidation (Figure 9), as ROS levels did not decrease in either the UFAO or the HF-HC+UFAO groups, with respect to the CTRL and HF-HC groups, respectively (Figure 8); however, mitochondria from the HF-HC group treated with Fe^2+^ ion for the in vitro induction of lipid peroxidation exhibited increased lipid peroxidation resistance with respect to mitochondria from the CTRL group (Figure 9). The latter was to be expected given that benzofuranone-type compounds such as those found in UFAO (Compound **3**, Table 4) possess antioxidant activities several orders of magnitude higher than flavonoid compounds, from which some benzofuranones are derived [54]. In addition, as mentioned above, xanthine-type compounds such as astaxanthin, which are highly lipid soluble, accumulate in membranes where they are able to counteract lipid peroxidation but, consistent with our results, are not able to trap ROS produced in the ETC because they are emitted into the water-soluble environment of the matrix or intermembrane space [48]. Therefore, tangeraxanthin (i.e., compound **5**, Table 4), could act in this way, decreasing lipid peroxidation in the hydrophobic environment of mitochondrial membranes (Figure 9) but not in the hydrophilic environment where ROS are produced (Figure 8), explaining why there was no decrease in ROS production.

Another puzzling result is that liver mitochondria respiration in the UFAO group decreased significantly to the levels of the HF-HC group (Figure 3). This may be related to decreased complex I activity and marked inhibition of the complex II–complex III segment of the ETC in both liver and kidney mitochondria (Figure 7), causing less electron flow throughout the entire ETC and leading to lower oxygen consumption (Figure 3a). This, in turn, would increase ROS production by increasing the half-life of ubisemiquinone radicals formed at the redox sites of complexes I and III, resulting in an incomplete reduction of oxygen to superoxide anion. This would explain the ~2-fold increase in ROS production in liver and kidney mitochondria in the UFAO group (Figure 8). One of the limitations of this work is that we do not know the reason for this phenomenon, and we do not have experimental evidence to develop a hypothesis to explain it. 

Despite the somewhat discouraging results of the effect of UFAO on the activity of ETC complexes (Figure 6 and Figure 7), UFAO treatment in healthy rats (i.e., the UFAO group) did not statistically significantly alter the RCR values of liver and kidney mitochondria (Figure 3 and Figure 4), reflecting that the inhibition in the activity of ETC complexes was not directly proportional to a decrease in OXPHOS. In support of this notion, the *ΔΨ* of rat liver and kidney mitochondria was not appreciably decreased in the same group (Figure 5). Since *ΔΨ* is the driving force for ATP synthesis [55], the null effect of UFAO in healthy rats is consistent with a negligible effect of UFAO on OXPHOS. This warrants further study to determine whether UFAO causes deleterious effects at the tissue and organ level in healthy animals or whether this is offset by the minimal alterations in OXPHOS and *ΔΨ* observed in our results. 

Finally, the impact of HF-HC diets on lipid metabolism is well established, particularly their role in promoting dyslipidemia [8]. In our study, the HF-HC and HF-HC+UFAO groups showed elevated cholesterol levels compared to the CTRL group (Table 5). While treatment with UFAO did not result in a statistically significant reduction in cholesterol levels, a slight downward trend was observed in the HF-HC+UFAO group compared to the HF-HC group. This suggests that UFAO may exert a slight cholesterol-lowering effect, although potential benefits over a more extended treatment duration or in combination with other pharmacological interventions should be evaluated. Unexpectedly, the UFAO group exhibited higher triglycerides and VLDL levels. These results suggest that UFAO may have an impact on lipid metabolism in healthy rats; one possible mechanism could involve the bioactive components present in UFAO that might influence lipid metabolism pathways in a way that promotes lipid synthesis or inhibits lipid clearance in healthy rats. Further research is needed to elucidate the exact pathways and interactions involved. In contrast, HDL levels did not differ statistically among groups. However, a notably significant reduction in LDL levels in the HF-HC+UFAO group suggests UFAO protects against diet-induced dyslipidemia. This effect may result from the actions of bioactive compounds found in UFAO, which may inhibit cholesterol absorption and prevent LDL oxidation. These results suggest the use of UFAO as a potential dietary intervention to reduce LDL and cardiovascular risk.

## 5. Conclusions

Supplementation with UFAO improved glucose utilization and insulin resistance in rats consuming an HF-HC diet. This could be related to the enhanced oxidation of metabolic substrates in liver mitochondria and an antioxidant effect of UFAO without modifying ROS production. Furthermore, the beneficial effects of UFAO on mitochondrial function in the kidneys of rats on an HF-HC diet could imply a protective effect against renal lipotoxicity induced by this diet. Some of the limitations of this study are that we did not identify which components of UFAO are responsible for the beneficial effects observed in rats on the HF-HC diet. Also, we did not evaluate whether the effects of UFAO at the mitochondrial level translate into an improvement in the pathophysiological alterations that may occur at the hepatic and renal levels by the HF-HC diet. Finally, although the HF-HC diet induced insulin resistance, rats on this diet did not develop hyperglycemia; thus, it was not possible to determine whether UFAO regulates glucose levels in diabetes.

## Figures and Tables

**Table 1:** Composition of experimental diets.

Diet Components	Control	HF-HC
Calories (kcal)	440.5	501 *
Total Carbohydrates %	62	60
Fructose %	-	25
Sucrose %	-	10
Lactose%	-	10
Total Fat %	8.5	21
Saturated fat %	-	8
Trans fat %	-	3.6

* Calories of solid food, not including fructose.

**Table 2:** Fatty acid composition of avocado oil (chosen foods S.A de C.V).

Fatty Acid	Relative Proportion (%)
Palmitic acid	15.7
Palmitoleic acid	5.4
Esteraric acid	1.3
Oleic acid	59
Linoleic acid	16.5

Main fatty acids identified by GC-MS are shown.

**Table 3:** Saturated aliphatic compounds present in UFAO ^1^.

Compound Name	Relative Proportion (%)
Decane	7.65
4-Methyldecane	2.87
2-Ethyl-1-hexanol	6.68
2-Cyclohexyldecane	1.89
(E)-Tridec-2en-1-ol	4.05
Undecane	19.55
1-Methyldecahydronaphthalene	4.65
6-Methylundecane	1.44
2-Decanone	4.56
Dodecane	17.19
2,6-dimethylundecane	6.61
2,3,7-Trimethyloctane	4.79
Tridecane	11.85
Tetradecane	2.82

^1^ Identification of compounds was carried out by GC-MS.

**Table 4:** High-molecular-weight compounds in UFAO ^1^.

Compound Number	Compound Name
**1**	(2*E*,4*E*)-3,7-Dimethyl-6-(5,5,8,8-tetramethyl-5,6,7,8-tetrahydro-naphthalen-2-yl)-octa-2,4,6-trienoic acid
**2**	Gluroside
**3**	Loliolide β-D-glucopyranoside
**4**	Yahyaxanthone
**5**	Tangeraxanthin
**6**	[(3*R*,4*S*,5*R*,6*R*)-3-dodecanoyloxy-2-ethoxy-5-hydroxy-6-(hydroxymethyl)oxan-4-yl] dodecanoate

^1^ Identification of compounds was carried out by ESI (negative and positive modes).

**Table 5:** Effect of UFAO on fasting serum lipid profile ^1^.

Biochemical Parameters	CTRL	UFAO	HF-HC	HF-HC+UFAO
Total cholesterol (mg/dL)	91.3 ± 10.4 ^b^	83.7 ± 3.9 ^b^	165.8 ± 14.3 ^a^	157.2 ± 20.6 ^a^
Triglycerides (mg/dL)	43.0 ± 7.2 ^b^	73.7 ± 17.6 ^b^	39.3 ± 1.5 ^a^	37.4 ± 5.6 ^a^
HDL (mg/dL)	79.7 ± 8.4 ^a^	65.2 ± 5.1 ^a^	68.3 ± 21.8 ^a^	89.6 ± 16.5 ^a^
LDL (mg/dL)	27.9 ± 10.3 ^b^	35.9 ± 3.8 ^b^	115.9 ± 23 ^a^	54.5 ± 6.7 ^a^
VLDL (mg/dL)	8.3 ± 1.5 ^b^	14.7 ± 3.5 ^b^	7.9 ± 0.3 ^a^	7.5 ± 1.1 ^b^

^1^ Values represent the mean ± standard error of *n* = 4. Statistically significant differences between means were determined with one-way ANOVA and Holm–Sidak post hoc test. Different letters (a, b) indicate statistical differences (*p* < 0.05) between groups in each biochemical parameter.

## Data Availability

The raw data supporting the conclusions of this article will be made available by the authors on request.

## Supplementary Materials

The following supporting information can be downloaded at: https://www.mdpi.com/2218-1989/14/8/431/s1, Figure S1: Chemical structure of high-molecular-weight compounds in UFAO.
