# Supplementary material for: Evaluation of Unsaponifiable Fraction of Avocado Oil on Liver and Kidney Mitochondrial Function in Rats Fed a High-Fat and High-Carbohydrate Diet
Source: Metabolites. 2024 Aug 4;14(8):431. doi: 10.3390/metabo14080431 (PMC11487379; doi:10.3390/metabo14080431)
Supplement: Supplementary file 1 [file metabolites-14-00431-s001.zip › metabolites-3112588-supplementary.pdf]

Supplementary Figure S1. Chemical structure of high molecular weight compounds in UFAO .

Molecular weight	Name	Structure
351.2291	(2*E*,4*E*)-3,7-Dimethyl-6-(5,5,8,8-tetramethyl-5,6,7,8-tetrahydro-naphthalen-2-yl)-octa-2,4,6-trienoic acid	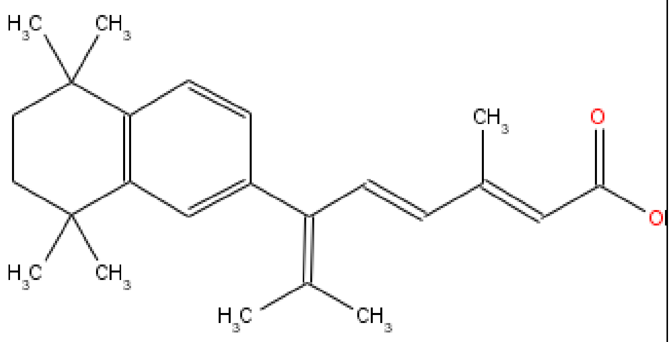
333.1565	Gluroside	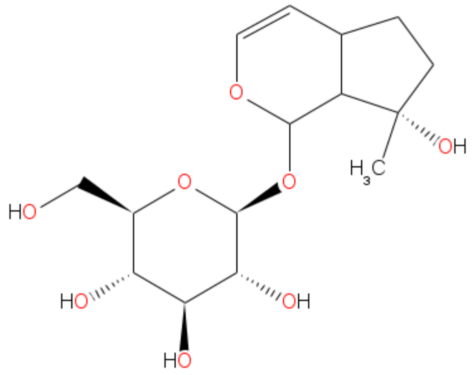
359.1921	Loliolide β-D-glucopyranoside	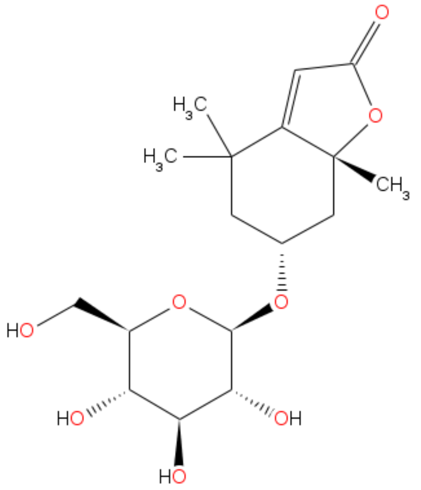

415.137	Yahyaxanthone	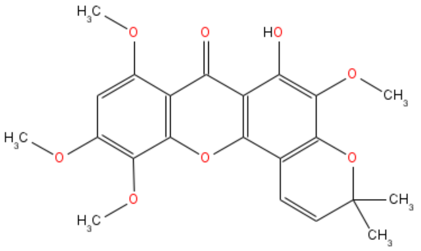
485.3381	Tangeraxanthin	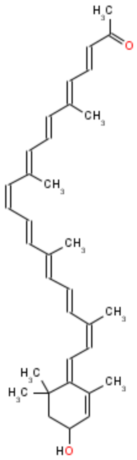
573.4388	[(3*R*,4*S*,5*R*,6*R*)-3-dodecanoyloxy-2-ethoxy-5-hydroxy-6-(hydroxymethyl) oxan-4-yl]dodecanoate	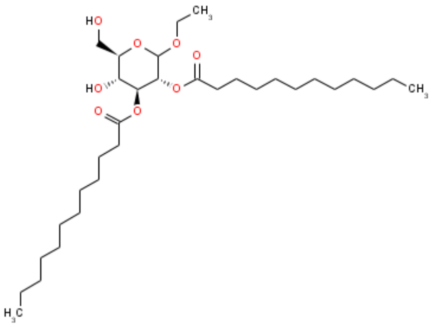
